# Effects of Different Staircase Design Factors on Evacuation of Children from Kindergarten Buildings Analyzed via Agent-Based Simulation

**DOI:** 10.3390/healthcare8010056

**Published:** 2020-03-09

**Authors:** Jiaxu Zhou, Xiaohu Jia, Junhan Jia

**Affiliations:** 1 Architecture College, Inner Mongolia University of Technology (IMUT), Hohhot 010051, China; jiaxuzhou@126.com; 2 Inner Mongolia Engineering Technology and Research Center for Green Buildings, Architecture College, Inner Mongolia University of Technology, Hohhot 010051, China; 3 Inner Mongolia Key Laboratory of Green Building, Architecture College, Inner Mongolia University of Technology, Hohhot 010051, China; 4 Beijing Aidi School, Beijing 100000, China; junhanjia@126.com

**Keywords:** staircase design methods, children evacuation, evacuation simulation, agent-based model, evacuation time, evacuation efficiency

## Abstract

Staircase design is critical to the evacuation of children. Through an agent-based simulation, this study focused on the relationship between staircase design factors and evacuation efficiency in a multi-story kindergarten. A quantitative study was conducted on three critical architectural design factors: stair flight width, positional relationship, and design pattern of the juncture between the staircase and the corridor. The findings were as follows. (1) When the stair flight width ranges from 0.7 to 1.0 m, an increase in this width can improve evacuation efficiency significantly; when the width ranges from 1.1 to 1.4 m, evacuation efficiency is improved continuously, but an increase in this width range has a diminishing effect on evacuation efficiency; when the width is greater than 1.7 m, a further increase has an adverse effect on evacuation efficiency, because such a staircase space allows overtaking behaviors. (2) Under the same stair flight width conditions, evacuation efficiency is higher when the staircase and corridor are perpendicular to each other than when they are parallel, because the natural steering angle of the children was preserved during their evacuation. (3) The cut corner and rounded corner designs between the staircase and corridor improved evacuation efficiency and alleviated the congestion at bottleneck positions; the evacuation efficiency continued to rise with an increase in the cutting angle. These findings are expected to provide a useful reference for the evacuation design of kindergarten buildings and for emergency evacuation management.

## 1. Introduction

China is a populous country, in which there are now more than 220 million children aged between 0 and 14 years [1]. However, few studies have focused on the evacuation of children from kindergartens. Since the implementation of China’s two-child policy, families with two children have become a common occurrence in China; as a result, the Chinese population that needs to be educated on this topic continues to increase. Because of the shortage of urban land, multi-storied instead of single-storied kindergarten buildings are being constructed in China. Active development of fundamental education has been a major strategy in China in recent years. Considering the frequent occurrence of safety accidents, the safety of kindergarten buildings has become a common subject of concern in Chinese society [2,3]. 

Panic is defined as a highly nervous state arising from a state of emergency; in a panic state, people crowd against each other for their own safety, and a force of at least 4450 *N* is generated between the people; this force is sufficient to cause casualties [4]. The fire that occurred at the Artistic Kindergarten of Jiangxi Radio & Television Development Centre is a noticeable example. During the fire, 540 children rushed to an escape exit simultaneously, causing deaths of 13 children (including seven boys and six girls). It was noted that the casualties were possibly not caused by direct hazards (e.g., burning), but by a severe stampede in the evacuation process due to over-crowdedness [5]. In the Genghe Community Kindergarten in Zhengzhou City, Henan Province, a large crowd of people rushed through a single passageway (i.e., corridor and staircase), and a bottleneck was formed in the evacuation process; because of the low evacuation efficiency, three children were unable to escape and lost their lives [6]. In a kindergarten on Qinling Road, Zhengzhou City, a similar accident occurred; many children were injured but, fortunately, no deaths occurred [7]. Regardless of the real hazards, the above cases were similar. Even if staff members help children evacuate, highly disordered children fill up the limited space, causing high risks of congestion, stampede accidents, and possible severe casualties. Focusing on evacuation design in a kindergarten, this study discussed the relationship between staircase design factors and evacuation efficiency. The findings of this study provide a useful reference for the evacuation design of kindergarten buildings and for emergency evacuation management. 

In recent years, computer simulation has been the main method used for studying personnel evacuation [1,2], and it plays an important role in predicting pedestrian behaviors in the evacuation process, as well as in evacuation design. Worldwide, researchers from different disciplines have proposed diverse evacuation models. Using the modeling approach, personnel evacuation models can be classified roughly into macro models and micro models. Macro-modeling involves regarding pedestrians as a continuous flowing medium; modern studies on personnel evacuation are based on studies of traffic flow, and so they inherit the mature research approach used in fluid studies. Henderson et al. [8] proposed the first macro evacuation model and argued that pedestrians’ motion behaviors are similar to the flow of gas or liquid; moreover, the gaseous dynamics equation describing pedestrian behaviors is similar to the Boltzmann equation, except that it considers the interactions of pedestrians and the purpose of these interactions. In light of the continuum theory, Hughes et al. [9,10] studied the motion characteristics of large crowds; using the Navier–Stokes equation, the authors further deduced the control equation for the flow of large crowds, as well as the equation for preventing pedestrians from moving toward dense crowds. The macro model successfully accounted for the motion status of the crowds during the pilgrimage to Mecca. However, macro evacuation models do not consider the interactions of people, and so they are not suitable for the study of pedestrian flow in emergency circumstances. Macro evacuation models ignore the differences between individuals; hence, researchers proposed a new type of evacuation model, namely, micro evacuation models. Micro evacuation models regard pedestrians as interacting particles, and they can describe the specific behaviors of pedestrian flow. In recent years, micro-evacuation models have received much attention. These models can be classified into discrete evacuation models and continuous evacuation models. The representative micro evacuation models include social force (SF) [11], agent-based (AB) [12], cellular automaton (CA) [13], and lattice gas (LG) models [14]. However, CA or LG models only scarcely emphasize behavioral characteristics [15]. AB models have bright application prospects; fundamentally, individuals in AB models are viewed as self-thinking agents who can perceive and react to the surrounding environment. By setting parameters that influence human actions, AB models can simulate the evacuation behaviors of humans in real scenarios, thus obtaining simulated data [16]. The representative software of AB models includes Simulex (Integrated Environmental Solutions. Ltd (IES), London, UK) [17], EXIT89 (National Fire Protection Association (NFPA), Quincy, MA, USA) [18], and EXODUS (Fire Safety Engineering Group (FSEG), Greenwich, UK) [19]. 

Considering the advantages of simulation models, many scholars have used them to study the evacuation problem in recent years. Helbing et al. [20] proposed an SF model for single-exit and double-exit rooms to investigate various effects (e.g., arch effect, congestion effect, bandwagon effect, and amplitude-based effect) in the evacuation process. Using a CA model, Perez et al. [21] investigated the behavioral characteristics of personnel flow under different exit conditions. Using an extended CA model, Kirchner et al. [22] studied the friction effect and congestion phenomenon. Fang et al. [23] improved the biased random walk model, and investigated the exit choice behaviors of people accordingly. Nagai et al. [24] studied the dynamic characteristics of personnel evacuation within the sphere of zero vision. Isobe et al. [25] studied the evacuation process and bandwagon effect of people within the sphere of zero vision. Toyama et al. [26] studied people’s bandwagon behaviors and obstacle-avoiding behaviors. Song et al. [27] proposed a multi-grid model in which the effect of force was considered. Wu et al. [28] proposed a CA model in which auditory perception was considered, to investigate the effects of auditory factors in the process of large-space evacuation. Using an affordance-based finite-state automaton model, Joo et al. [29] conducted a simulation study, in which adult perception was considered in the evacuation models. 

The above studies revealed essential characteristics of the evacuation process, providing useful reference for organizers or managers with respect to personnel evacuation. Some findings or conclusions can be applied directly to elements of architectural design (e.g., corridors, exits, stairs, and other spaces). Using an AB model, Li et al. [30] simulated the process of personnel evacuation in a four-story office building. Lo et al. [31] simulated an evacuation process during which personnel pass through a corridor, and found that corridor width affects the overall evacuation time; specifically, the evacuation time decreased as corridor width increased. However, evacuation time remained constant when the exit width reached a specified critical value. With the help of software, Chow et al. [32] analyzed the effects of passageway width and exit setup on the process of personnel evacuation in a building typical of a karaoke company. In a fire-free conventional evacuation process, however, the results obtained through numerical simulation showed that an increase in corridor width does not necessarily reduce the evacuation time. Shende et al. [33,34] studied both the optimal evacuation process, in which personnel evacuated to a spacious area through a corridor passageway, and the flowing speed of pedestrians in a corridor passageway. Qu et al. [35] investigated the effects of the geometric dimensions of stairs in subway stations, step restrictions of staircases, and motion speed on personnel evacuation. Using evacuation simulation software, Ding et al. [36] studied how to evacuate personnel via a combination of stairs and elevators in high-rise buildings. Sun et al. [37] through a simulation of mass evacuation, explicated the pattern of impact between planning form of residential areas and the efficiency of evacuation, proposing strategies and methods for optimizing the existing structure of community road network. 

The above studies on evacuation simulation provide a basis for the study of pedestrian dynamics. However, although these studies concentrated on the mechanism of pedestrian dynamics, they did not try to extend their findings or conclusions. Therefore, there have been no studies on evacuation at a practical level. At a practical level, existing studies on evacuation have mainly focused on high-rise buildings [38], transportation buildings [39], and gymnasia [40]. However, such studies have paid very little attention to kindergarten buildings. Because of the special nature of kindergarten buildings, some universal research findings cannot be used directly to guide their evacuation design. Using an agent-based model, this study assessed ways to improve evacuation efficiency through the appropriate staircase design of a kindergarten, including the width of the stair flight, positional relationship between the staircase and corridor, and design pattern of the juncture between the staircase and corridor. As a scientific basis, the findings of this study can contribute to the architectural design of kindergarten buildings. 

## 2. Model Formulation

### 2.1. Sample Collection 

**Figure 1:**
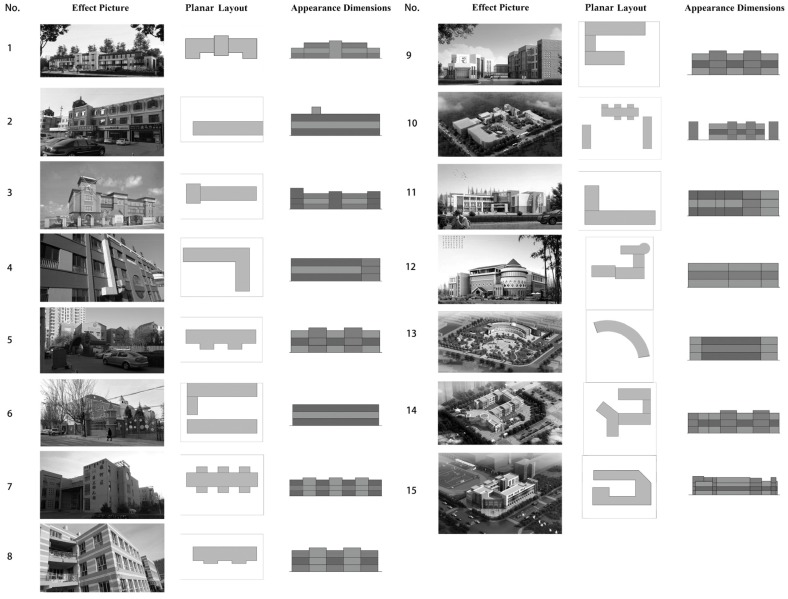
Summary of the kindergartens’ physical space.

In this study, we investigated 15 kindergartens in China’s Inner Mongolia, a typical example of a severely cold region in China. All data used in this study were acquired through field measurement, from the relevant authorities, or from construction drawings of the institutions. The investigation content mainly covered the physical environment built in the spatial model and the behavioral properties of the personnel to be evacuated, including two types of elements. For the spatial model, the investigated items included the general layout, plane layout, and appearance dimensions of the kindergartens (as described in Table 1 and Figure 1); for the behavioral properties, the investigated items included the daily life patterns of the children. The investigation results are summarized as follows.

**Figure 2:**
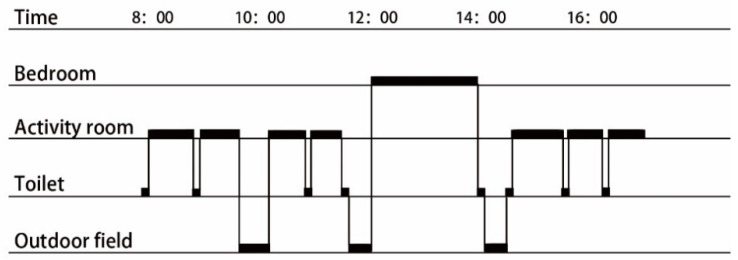
Daily life routines of the kindergartens.

**(1)**  **General layout**: Considering the planar function arrangement and required conditions for the growth and development of children, a tree-shaped layout is employed (the single-line layout also fell under the tree-shaped layout), and the living units are listed separately. This is beneficial for functional division and allows for an appropriate space for outdoor activity.
**(2)**  **Planar form**: The studied kindergartens are all located in a severely cold region, and a one-way corridor-type layout is employed for them. In terms of the planar form, the buildings mostly have a linear layout. This is beneficial for providing favorable sunshine and ventilation and for promoting the growth and development of children.
**(3)**  **Appearance dimensions**: The teaching areas are usually located below the second floor, and some offices are located on the third floor.
**(4)**  **Daily life pattern**: Unlike primary school education, early childhood education is not dominated by classroom teaching, but it is designed to arrange various activities according to the life management regulations. Whether certain teachers or children are scheduled to attend activities is based on the life management regulations, and children attend various centralized or decentralized activities according to the specified order of life. The cerebral cortex can develop a conditioned reflex through long-time repetitive stimulation, thus protecting the normal neural development of children. Figure 2 summarizes the daily life routines of the 15 kindergartens.
  (Note: In China, a living unit comprises activity rooms, bedrooms, toilets, and cloakrooms.)

### 2.2. Building Model 

The above analysis of the investigated sample showed that the children’s daily activities were usually completed within the living units. 

**Figure 3:**
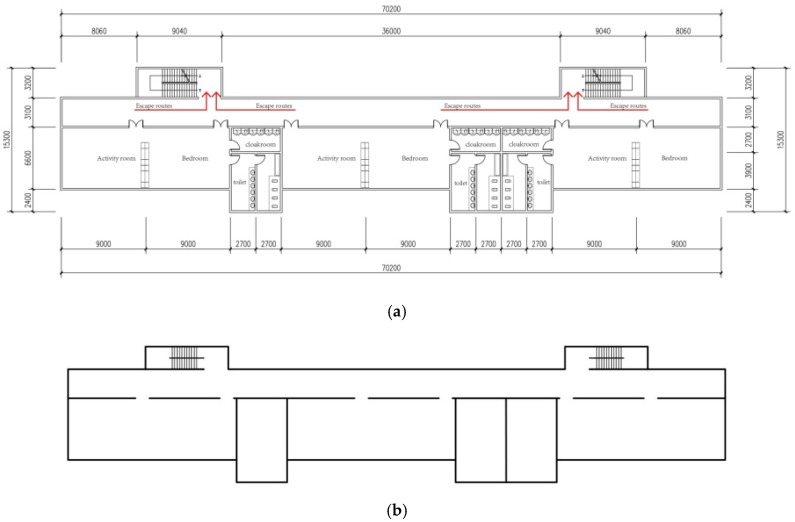
(**a**) Basic planar model; (**b**) simplified basic planar model.

In the basic model for this study, a basic planar model is built according to the functional body (i.e., living unit) of each kindergarten. Figure 3a shows the details and evacuation routes of the basic planar models. To simplify the redundant spatial information and improve the efficiency of the computer simulation, the basic planar model was simplified (as shown in Figure 3b). Accordingly, this study analyzed the effects of various staircase design factors on evacuation efficiency. The related dimensions and area complied with China’s *Code of Building Design for Nurseries and Kindergartens* (JGJ 39-2016) and the *Code for Fire Protection Design of Buildings* (GB50016-2014).

1. The children’s activity places (e.g., children’s room and recreation hall) in a nursery or kindergarten should be set up in detached buildings, but not underground or semi-underground. The detached buildings should not be more than three-storied if their fire resistance comes up to Grade 1 or 2.
2. The living unit in a kindergarten should meet the following requirements for minimum usable area: 70 m^2^ for an activity room, 60 m^2^ for a bedroom, 12 m^2^ for a toilet, 8 m^2^ for a washroom, and 9 m^2^ for a cloakroom.
3. For an activity room with one-sided daylight, the depth should not be more than 6.60 m.
4. For the stairs used for children, the stair step height should be 0.13 m, and the stair step width should be 0.26 m.
5. For the living unit of a kindergarten, the width of a one-sided corridor should be more than 1.8 m.
6. For a room located between two emergency exits, the maximum distance between the door and the nearest external exit or staircase should be 25 m; for a room located at two sides or at the end of a bag-shaped corridor, the maximum distance between the door and the nearest external exit or staircase should be 20 m.

### 2.3. Building Occupants

In the evacuation model, three circles were used to denote the planar shapes of evacuees. The middle of an agent was represented a large circle, with two small circles like shoulders. Actual evacuees were simulated accurately. In the evacuation process, the significant difference between children and adults lies in their size dimensions [3]. To determine the dimension parameters of the agent, appropriate tape types were selected to model the body size of children. 

**Figure 4:**
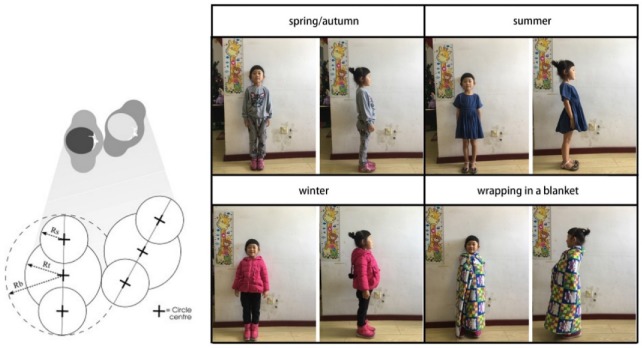
Size of agent and children’s clothing in different seasons.

In this study we considered the dressing of children in different seasons to determine the body sizes of children. The clothes worn by children vary from season to season. As shown in Figure 4, this study considered four scenarios of dressing (i.e., wearing indoor clothes in spring and autumn, wearing clothes in summer, wearing indoor clothes in winter, and wearing blankets or a full set of warm clothes). A child with an average body size was selected to investigate the four scenarios and calculate the average values in the scenarios, as described in Table 2. In this evacuation model, the evacuation speed of children was determined based on experimental data regarding the evacuation of Chinese preschool children; to be more specific, the movement speed on a horizontal surface was 0.81 ± 0.40 m/s, and the movement speed on a staircase was 0.63 ± 0.20 m/s [1]. In this evacuation model, the children’s movement speed on the horizontal surface was 0.90 ± 0.30 m/s, and their movement speed on a staircase was equal to half of that on a staircase. This showed that children’s body size and movement speed were highly consistent with previous experimental data. Therefore, the type of occupant was set to “children”. 

Occupants were distributed on the first floor and second floor. There were 24 children in each class, a total of six classes on the first and second floors, and a total of 144 children to be evacuated. The “tape” type was set to “all children”. Each classroom had two exits, and the exit width was 1.2 m. Child positions were averagely distributed according to their density. In a real evacuation scenario, this method can be used to maintain the initial positions of personnel well and to ensure appropriate occupancy of indoor space. Table 3 describes the specific modeling parameters. It was assumed that all agents responded promptly at the start of each scenario. Therefore, response time was not used as a parameter. 

### 2.4. Simulation Modeling

In this study, a Simulex 4.0 agent-based model (a microsimulation model) was used as a simulator to reflect the movement law of evacuees. Table 4 describes the specific modeling details. In the evacuation process, evacuees selected an exit along the shortest evacuation route. The simulation model was divided into a number of 0.2 × 0.2 m grids, each of which was connected with 16 nodes. For complex building simulation, hundreds of thousands of nodes are sufficient to denote the positions of obstacles and evacuees inside buildings accurately [17]. 

The simulation system supported the floor plans of multiple stories and the simulation of evacuation by multi-story stairs, and it directly imported the DXF file from the AutoCAD software. It was able to simulate the evacuation of tens of thousands of people in a complex building environment, record the movement process of evacuees, and calculate the number of evacuated personnel at each exit at regular intervals of 5 s. 

**Figure 5:**
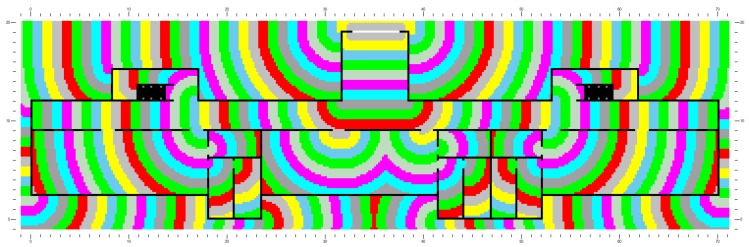
The distance map.

In the simulation system used in this study, a building comprises four parts: floor, staircase, link, and exit. A link refers to the connection between a staircase and a floor. In an evacuation model, personnel enter the staircase from a floor or enter a floor from the staircase. When evacuees pass through an exit, the evacuation process is complete. The distance map is used to describe the moving distance from any point inside the building to an exit. Various color bars denote the variation in optimal distance (unit: m), as shown in Figure 5. 

It should be noted that by denoting the interactions as being between children, the model was able to describe the evacuation process according to the behavioral characteristics of children. To reflect the actual evacuation process, the interactions included free motion, queuing, overtaking, rotation, and collision avoidance. When the density of evacuees was less than two evacuees per m^2^, evacuees slowed down and redirected their movement if blocked by other evacuees ahead; alternatively, evacuees tried to overtake other evacuees if permitted by space. When evacuees converged to a narrow passageway, they queued up. In the evacuation process, the speed of evacuees’ movement varied according to the evacuee interval and obstacle-free walking speed (*Vu*); the relationship between evacuation speed (*v*) and evacuee interval (*d*) can be expressed by Equation (1) [17].

(1) $$v=\{\;\begin{array}{c} V_{u}\times sin\left[90\times \left(\frac{d-b}{t_{d}-b}\right)\right],b\leq d\leq t_{\begin{array}{c} d \end{array}}\\ V_{u}\;,\;d>t_{d} \end{array}$$

where

v = obstructed walking speed (unit: $m\cdot s^{-1}$);
$V_{u}$ = obstacle-free walking speed (unit: $m\cdot s^{-1}$);
*D* = evacuee interval (unit: m);
$t_{d}$ = interval threshold; and
*b* = body thickness.

**Figure 6:**
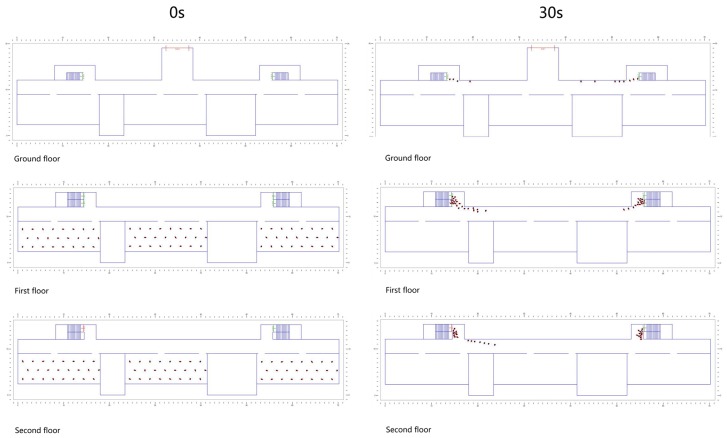
Snapshot of the evacuation simulation.

Through numerical simulation, we recorded the time during which all evacuees escaped to exits through the staircases. We analyzed their evacuation time comparatively, thus investigating the effects of different staircase design factors (including the size of stair flight, positional relationship between the staircase and corridor, and design pattern of the staircase antechamber) on evacuation efficiency. Figure 6 shows a snapshot of the evacuation simulation using an agent-based model. 

## 3. Simulation Results and Discussion 

### 3.1. Effects of Width of Stair Flight on Evacuation Efficiency 

**Figure 7:**
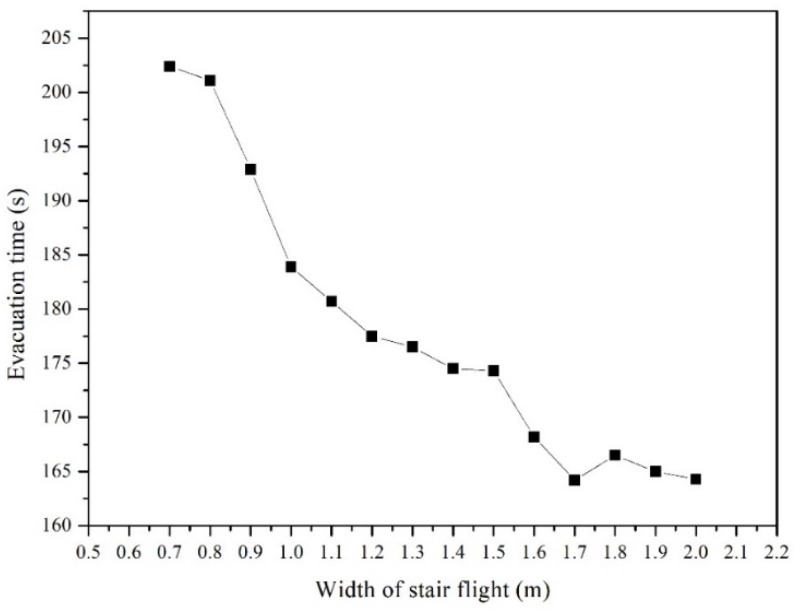
Relationship between the width of stair flight and evacuation time.

In this simulation, all the personnel were evacuated along the shortest routes. The evacuation time was defined as the time required to evacuate all personnel via the exits on the ground floor. To reduce the deviation of simulation results arising from other variables, the stair step height was 0.13 m, stair step width was 0.26 m, and each stair flight comprised 15 steps. In addition, we considered stair flights with widths of 0.7 m, 0.8 m, 0.9 m…1.9 m, and 2.0 m. The intent was to determine the effects of the width of the stair flight in a kindergarten building on evacuation efficiency. Each group of parameter settings was used to repeat the simulation process 30 times to obtain simulated results of average evacuation time, as described in Table 5. After the average evacuation time was determined, we selected the simulation results with the same average value and generated a diagram of the relationship between evacuation time and width of stair flight, as shown in Figure 7. 

As shown in Figure 7, when the width of the stair flight increased from 0.7 m to 1.0 m, evacuation time decreased significantly, implying a significant improvement in evacuation efficiency. In particular, the evacuation time decreased by 4.1% and 4.7%, respectively, when the width of stair flight increased from 0.8 m to 0.9 m and from 0.9 m to 1.0 m. This implies that an increase in the width of stair flight within this range improved evacuation efficiency significantly. The evacuation time decreased by 1.7% and 1.8%, respectively, when the width of stair flight increased from 1.0 m to 1.1 m and from 1.1 m to 1.2 m. However, evacuation time decreased slowly when the width of stair flight increased from 1.2 m to 1.5 m. Evidently, the width of the stair flight in the range of 1.2 m to 1.5 m had no significant effects on evacuation efficiency. In addition, evacuation time decreased by 5.8% when the width of stair flight increased from 1.5 m to 1.7 m, but increased rather than decreased when the width increased from 1.7 m to 2.0 m. Evidently, the width of stair flight had a negative effect on evacuation efficiency when it was greater than 1.7 m. This is because the larger space in the staircase allows overtaking behaviors in the evacuation process. Subsequently, the force of friction increases due to collision and squeezing between different evacuees at the exits of passageways; this increases the force of competition at the exits, thus causing the phenomenon of “faster is slower” [41]. Considering the physiological development and cognitive level of kindergarten children, the above behaviors are more prone to causing safety accidents. 

**Figure 8:**
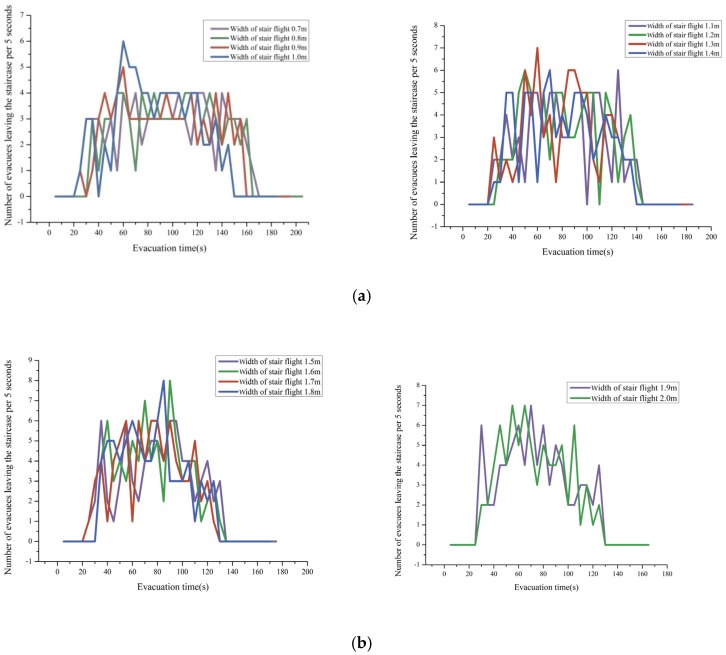
(**a**) Number of evacuees leaving the staircase per 5 s, Under the conditions of the stairs width of stair flight 0.7–1.0 m and 1.1–1.4 m; (**b**) number of evacuees leaving the staircase per 5 s, Under the conditions of the width of stair flight 1.5–1.8 m and 1.9–2.0 m.

Figure 8a,b shows the number of evacuees leaving the staircase at regular intervals of 5 s under different widths of stair flight. The evacuation process is divided into three stages: stage of evacuee increase, stage of evacuee stability, and stage of evacuee decrease. 

When the width of stair flight was 0.7 m to 1.0 m and 1.1 m to 1.4 m, the three stages of the evacuation process were as follows: (1) In the first stage, the number of evacuees increased rapidly to the maximum value; this stage accounted for approximately 20% of the total evacuation time. (2) In the second stage, the number of evacuees tended to be stable, implying a good order of evacuation; this stage accounted for approximately 60% of the total evacuation time. (3) In the third stage, the number of evacuees decreased rapidly until the end of the evacuation; this stage accounted for approximately 20% of total evacuation time. When the width of stair flight was 0.7 m to 1.0 m, the evacuation efficiency was four evacuees per 5 s in the stable stage, and the peak evacuation efficiency was six evacuees per 5 s. When the width of stair flight was 1.1 m to 1.4 m, the evacuation efficiency was as high as five evacuees per 5 s in the stable stage. 

When the width of stair flight was 1.5 m to 1.8 m, the peak evacuation efficiency reached 8 evacuees per 5 s, but the stage of evacuee stability accounted for only 20% of the total evacuation time. Evidently, there were competitive behaviors in the evacuation process. Sufficient space allows the occurrence of overtaking behaviors, thus shortening the stage of evacuee stability. When the width of stair flight was 1.9 m to 2.0 m, the number of evacuees increased first and then decreased, and the peak evacuation efficiency was seven evacuees per 5 s; moreover, the decrease in the number of evacuees was accompanied by a bounce, and there was no obvious stage of evacuee stability. This implies that competitive behaviors were more intense in the evacuation process. 

According to the simulation results, the number of evacuees was more stable, and the evacuation efficiency was higher when the width of stair flight was 1.1 m to 1.4 m. China’s *Code of Building Design for Nurseries and Kindergartens* (JGJ 39-2016) stipulates that the width of stair flight in nurseries and kindergartens should be greater than 1.1 m. Accordingly, the above results were further analyzed quantitatively. However, it is necessary to further verify the requirements for the width of stair flight specified in other codes of building design. 

### 3.2. Effects of the Positional Relationship between the Staircase and Corridor on Evacuation Efficiency 

**Figure 9:**
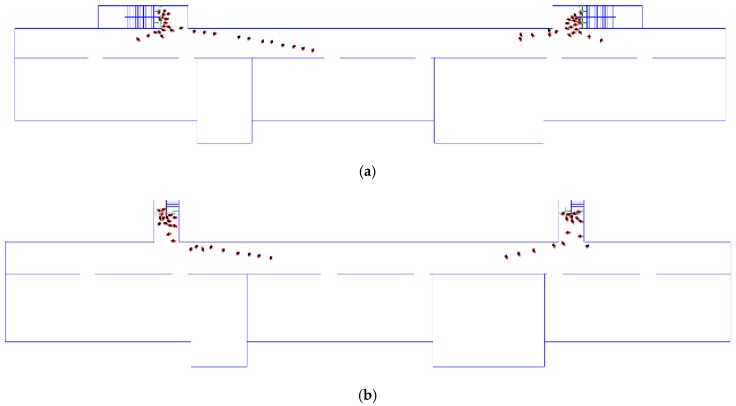
(**a**) Parallel, 1.2 m; (**b**) vertical, 1.2 m.

This stimulation considered the positional relationship (e.g., parallel or perpendicular) between the staircase and the corridor. Different design scenarios were simulated to investigate the effects of their positional relationship on evacuation efficiency. This evacuation model was built to improve the above model, keeping the width of stair flight as 1.2 m. When the staircase was perpendicular to the corridor and other parameter settings remained unchanged, the evacuation time shown in Figure 9a was compared with that shown in Figure 9b. 

**Figure 10:**
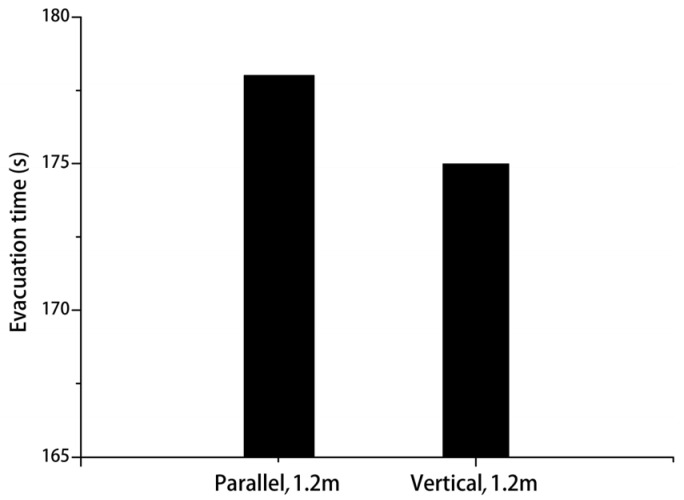
Evacuation time under different schemes.

**Figure 11:**
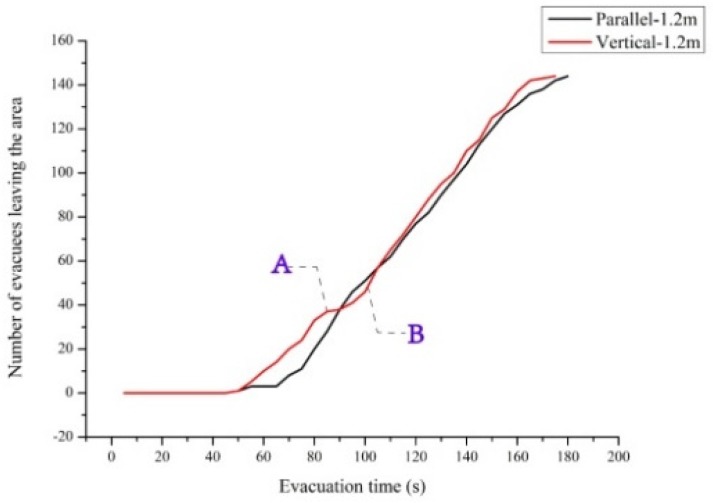
Relationship between the number of evacuees and evacuation time under different schemes. The process between points A and B represents the evacuation bottleneck.

The total evacuation time was 177.5 s when the staircase was parallel to the corridor, and it was 174.8 s when the staircase was perpendicular to the corridor—a decrease of 1.5%. Figure 10 shows the relationship between total evacuation time and the different schemes. Figure 11 shows the curve of the relationship between the number of evacuees and evacuation time in the evacuation process. Evidently, the overall variation trends of the number of evacuees and total evacuation time were consistent, regardless of whether the staircase was parallel or perpendicular to the corridor. However, the curve when they are perpendicular was overall above the curve when they were parallel. This implies that the overall evacuation efficiency was higher when they are perpendicular. It was not difficult to see that this trend was more obvious in the initial stage of evacuation. In the initial stage, when personnel are evacuating from the corridor to the staircase, a perpendicular relationship between the staircase and corridor reduces the angle required for the evacuees’ turn-around behaviors as compared with a parallel relationship between them. As a result, this decreases evacuation behaviors and improves the evacuation efficiency. In the middle stage of the evacuation, evacuees follow others due to the bandwagon effect. Therefore, the curves in the middle stage of evacuation were not as obvious as those in the initial stage. Overall, evacuation efficiency under the perpendicular relationship was higher than that under the parallel relationship. 

As shown in Figure 11, the curve under the perpendicular relationship had an inflexion point A, and the slope of the curve before A was higher than that after A. The slope of the curve denotes the flow of evacuees who pass through the staircase exit. After inflexion point A, there was a queuing phenomenon for a period. This was primarily due to improvement in the efficiency of evacuation from the initial positions to the staircase. After some personnel have evacuated successfully, evacuees on the first and second floors might be at the corner between the corridor and the staircase but might not have evacuated to the exit of the ground floor. After the evacuation process begins, there is a queuing phenomenon within a certain period (after inflexion point A). After most evacuees on the ground floor and first floor have evacuated successfully, the evacuation pressure at the bottleneck positions is low. Therefore, the slope of the curve became normal after point B. 

### 3.3. Effects of Design Pattern of the Juncture between the Staircase and Corridor on Evacuation Efficiency

**Figure 12:**
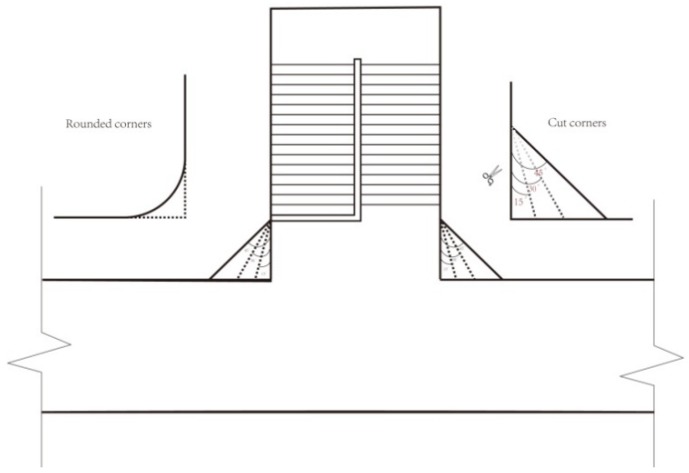
Right angle, cut (angle α: 15°, 30°, 45°), and rounded corner designs in the antechamber of the staircase.

As set forth above, the bottleneck position of evacuation is usually at the juncture between the staircase and the corridor. This simulation considered the three common design patterns of the critical bottleneck position. By simulating different design scenarios, this study investigated their effects on evacuation efficiency. Figure 12 shows the traditional design of the juncture between the corridor and the staircase, as well as the cut corner design at the juncture. The cut corner design involves the cutting angle α. This study then compared the effects on evacuation efficiency of the cutting angle α (15°, 30°, and 45°) and the rounded corner. 

**Figure 13:**
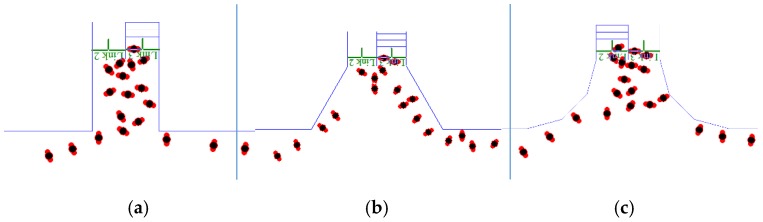
(**a**) Right angle design; (**b**) cut corner design; (**c**) round corner design.

In this evacuation simulation, the width of stair flight was the same as that in the above model (1.2 m), the staircase was perpendicular to the corridor, and the parameter settings were the same as those in the above model. Scenario 1 pertained to the traditional right angle design at the juncture (cutting angle α was 0°). Scenarios 2 to 4 pertained to the cut corner design at the juncture (α is 15°, 30°, and 45°). Scenario 5 pertained to the rounded corner design at the juncture. It is noteworthy that all spline curves had to be changed into arcs in the AutoCAD software after the round shape was imported into the simulation system. Nevertheless, some short arcs could not be input directly. In this case, arcs were regenerated by broken lines. The round shape in the simulation program was roughly the same as that in the AutoCAD software to ensure the accuracy of moveable space inside buildings. To obtain more accurate results, each group of parameter settings was used to repeat the simulation process 30 times, and the average evacuation time was calculated. In addition, the evacuation times in Figure 13a–c were compared. 

**Figure 14:**
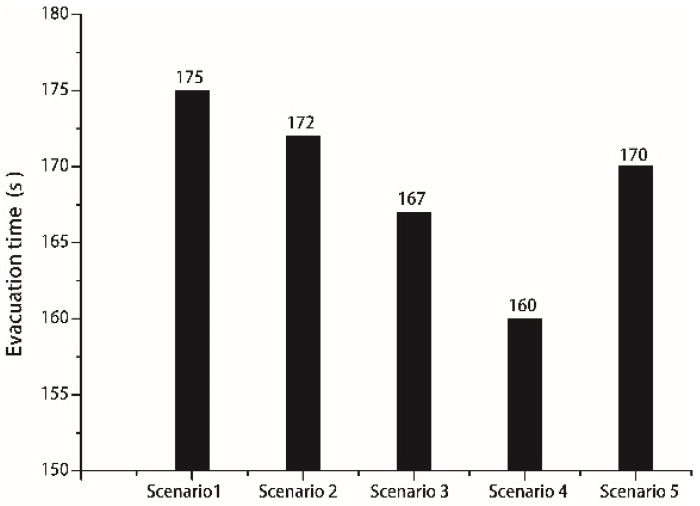
Relationship between total evacuation time and different scenarios.

Figure 14 shows the relationship between total evacuation time and different scenarios. Evidently, the evacuation times under the cut corner design and rounded corner design were shorter than that under the right angle design (Scenario 1). Compared with Scenario 1, cutting angles α of 15° (Scenario 2), 30° (Scenario 3), and 45° (Scenario 4) reduced the total evacuation time by 1.8%, 4.6%, and 8.6%, respectively. Compared with the right angle design (Scenario 1), the rounded corner design (Scenario 5) reduced the total evacuation time by 2.9%. Under the same conditions, the cut corner and rounded corner designs were beneficial to the evacuation process. When personnel evacuate from the corridor to the staircase, a cut corner design or rounded corner design allows the entry of more personnel. Moreover, the cut corner design avoids the inflexion point at the wall corner with a right angle. As a result, evacuees do not need to perform a sudden 90° turn-around behavior when they enter the wall corner, and they have a larger field of vision. When evacuees enter the next area, they can prejudge their upcoming behaviors properly, thus facilitating the whole evacuation process. In the subsequent motion, the narrowing wall space is more beneficial for leading the evacuees to move forward, thus alleviating the competitive behaviors between them. 

**Figure 15:**
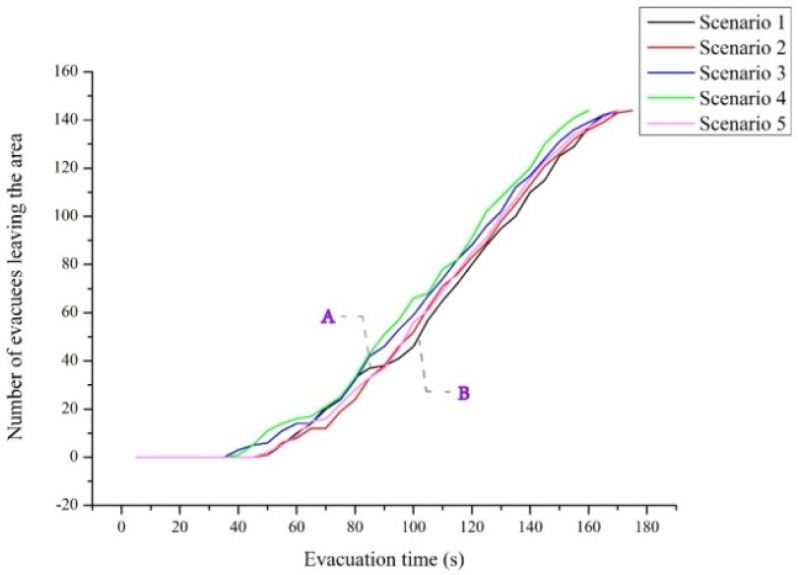
Relationship between the number of evacuees and evacuation time. The process between points A and B represents the evacuation bottleneck in Scenario 1.

Figure 15 shows the curve of the relationship between the total number of evacuees and evacuation time. The overall variation trends of the number of evacuees and evacuation time were consistent between the three design patterns. However, the curves under the cut corner and rounded corner designs were above the curve under the right angle design. Evidently, the two design patterns provided higher evacuation efficiency, which continued to improve with the increase in the cutting angle. 

Furthermore, there were no obvious inflexion points (i.e., A and B) in the curves under the cut corner and rounded corner designs. Evidently, the two design patterns alleviated the bottleneck and queuing phenomena at the juncture between the staircase and the corridor, thus facilitating the evacuation process. The design patterns of the small cutting angle and rounded corner were able significantly improve evacuation efficiency, possibly because children’s body sizes are smaller than those of adults. 

## 4. Conclusions

Through an agent-based simulation, this study quantitatively investigated the effects of three critical factors of staircase design in kindergarten buildings on evacuation efficiency. The investigated factors included the size of stair flight, positional relationship between the staircase and corridor, and design pattern of the juncture between the staircase and corridor, which is the critical position prone to generate a bottleneck during evacuation. The following conclusions were obtained through statistical analysis of simulation data. 

Through numerical simulation, this study analyzed and discussed the effects of width of stair flight on evacuation efficiency. We considered stair flights with widths of 0.7 m, 0.8 m, 0.9 m…1.9 m, and 2.0 m. Simulation results showed that evacuation efficiency is improved significantly when the width of stair flight was 0.7 m to 1.0 m. As the width of stair flight increased from 1.1 m to 1.4 m, the improvement in evacuation efficiency weakened. When the width of stair flight was greater than 1.7 m, its increase had a negative effect on evacuation efficiency; that is, the evacuation time increased. This was because the larger space inside the staircase allowed overtaking behaviors in the evacuation process, thus causing the phenomenon of “faster is slower”. In practice, this is more prone to cause accidents. According to the simulation results, the evacuation efficiency was higher when the width of stair flight is 1.1 m to 1.4 m. If a large-size staircase is designed to create a specific architectural form, it is recommended to add handrails at regular intervals of 1.1 m to 1.4 m. 

Moreover, this study investigated the effects of the positional relationship between the staircase and corridor on evacuation efficiency. We simulated the evacuation process under two common positional relationships (i.e., the staircase and corridor are perpendicular or parallel). When the width of the stair flight was the same, the evacuation efficiency under a perpendicular relationship was higher than that under a parallel relationship. 

For the bottleneck-prone critical position (i.e., the juncture between the staircase and corridor), this study discussed the effects on evacuation efficiency of three design patterns: (1) right angle, (2) cut corner by cutting the right angle, and (3) rounded corner. In addition, cutting angles α (e.g., 15°, 30°, and 45°) and rounded corners were simulated. The study found that the cut corner and rounded corner served to improve the evacuation efficiency and alleviate the congestion status at the bottleneck position, and the evacuation efficiency continued to improve with the increase in the cutting angle. 

Although this study successfully demonstrated the effect of different staircase design factors on children evacuation in kindergarten buildings by agent-based simulation, several limitations should be acknowledged. First, the effect of stair materials on children’s evacuation was not considered in this study, and we will investigate this issue in future research. Second, only architectural design factors were studied and analyzed in this study. Other relevant social factors, such as children’s behavior measured by on-site experiments, will be the subject of future research.

## Figures and Tables

**Table 1:** Name of kindergarten.

No.	Kindergarten
1	The Metallurgical Corporation of China, Shijia Branch of the Mongolian Kindergarten of Baotou City
2	Gold Age Kindergarten of Baotou City
3	Chenfeng Bilingual Kindergarten of Baotou City
4	Shuguang Kindergarten of Bayan Nur City
5	The No. 1 Kindergarten of Linhe District, Bayan Nur City
6	The No. 2 Kindergarten of Linhe District, Bayan Nur City
7	The No. 3 Kindergarten of Linhe District, Bayan Nur City
8	The No. 2 Kindergarten of Baotou City
9	Huifeng Kindergarten of Bayan Nur City
10	The No. 2 Kindergarten of Haibowan District, Wuhai City
11	The No. 3 Kindergarten of Haibowan District, Wuhai City
12	The No. 8 Kindergarten of Haibowan District, Wuhai City
13	Mongolian Kindergarten of Wuhai City
14	Yilin Kindergarten of Wuhai City
15	The No. 6 Kindergarten of Haibowan District, Wuhai City

**Table 2:** Specific sizes of clothes worn by children in different seasons and scenarios.

	Spring/Autumn 2Rb (m)	Summer 2Rb (m)	Winter 2Rb (m)	Wrapping in a Blanket 2Rb (m)	Average 2Rb (m)
Survey	0.342	0.298	0.476	0.562	0.4195
Simulex—children	/	/	/	/	0.420 ± 0.015

**Table 3:** Parameter settings for personnel.

No.	People	Parameter
1	Tape	All children
2	Total number of people	144
3	Ground floor: number of people	0
4	First floor: number of people	72
5	Second floor: number of people	72

**Table 4:** Parameter settings of the simulation model.

No.	Parameter	Quantity
1	Floor	3
2	Staircase	4
3	Link	8
4	Exit	1

**Table 5:** Width of stair flight and evacuation time.

**Width of stair flight (m)**	**0.7**	**0.8**	**0.9**	**1.0**	**1.1**	**1.2**	**1.3**
**Evacuation time (s)**	202.4	201.1	192.9	183.9	180.7	177.5	176.5
**Width of stair flight (m)**	**1.4**	**1.5**	**1.6**	**1.7**	**1.8**	**1.9**	**2.0**
**Evacuation time (s)**	174.5	174.3	168.2	164.2	166.5	165	164.3
